# Uptake of and factors associated with testing for sexually transmitted infections in community-based settings among youth in Zimbabwe: a mixed-methods study

**DOI:** 10.1016/S2352-4642(20)30335-7

**Published:** 2021-02

**Authors:** Kevin Martin, Ioana D Olaru, Nyasha Buwu, Tsitsi Bandason, Michael Marks, Ethel Dauya, Joice Muzangwa, David Mabey, Chido Dziva Chikwari, Suzanna C Francis, Mandikudza Tembo, Constancia Mavodza, Victoria Simms, Constance R S Mackworth-Young, Anna Machiha, Katharina Kranzer, Rashida A Ferrand

**Affiliations:** a Department of Global Health and Infection, Brighton and Sussex Medical School, Brighton, UK; b Clinical Research Department, London School of Hygiene & Tropical Medicine, London, UK; c Department of Infectious Disease Epidemiology, London School of Hygiene & Tropical Medicine, London, UK; d Department of Public Health and Policy, London School of Hygiene & Tropical Medicine, London, UK; e Biomedical Research and Training Institute, Harare, Zimbabwe; f AIDS and TB Unit, Ministry of Health and Child Care, Zimbabwe; g Division of Infectious and Tropical Medicine, Medical Centre of the University of Munich, Munich, Germany

## Abstract

**Background:**

The prevalence of sexually transmitted infections (STIs) among youth is high in sub-Saharan Africa. We investigated the uptake of testing for and prevalence of *Chlamydia trachomatis* (chlamydia) and *Neisseria gonorrhoeae* (gonorrhoea) infections among youth in community-based settings in Zimbabwe, and explored the facilitators and barriers to testing.

**Methods:**

This study was nested within a cluster randomised trial of community-based delivery of integrated HIV and sexual and reproductive health services for youth aged 16–24 years. Chlamydia and gonorrhoea testing via urine samples using the Xpert CT/NG test was offered in the four intervention clusters in Harare, Zimbabwe. Factors associated with testing uptake were investigated in a subset of participants (n=257) using hierarchical multivariate logistic regression. In-depth interviews with a separate purposively selected sample (n=26) explored facilitators and barriers to STI testing and partner notification and were analysed using thematic analysis.

**Findings:**

Between June 1, 2019, and Jan 31, 2020, there were 6200 attendances by 4440 participants (78·2% women, 21·8% men) median age 20·3 (IQR 17·9–22·8) years. 1478 participants had 1501 tests done, and 248 tests were positive and 1253 tests were negative for chlamydia or gonorrhoea, or both. STI test uptake was 33·3% (95% CI 31·9–34·7), increasing from 11·7% in June, 2019, to 37·1% in January, 2020. The prevalence of chlamydia or gonorrhoea, or both, was 16·5% (95% CI 14·7–18·5; 248 of 1501), with only seven participants (3%) showing symptoms. The overall yield of testing was 4·0% (95% CI 3·5–4·5; 248 of 6200). Uptake was associated with having symptoms (adjusted odds ratio [OR] 14·8, 95% CI 1·66–132·07) and negatively associated with being single (adjusted OR 0·33, 95% CI 0·13–0·84) or having a boyfriend or girlfriend (adjusted OR 0·19, 95% CI 0·087–0·43) compared with being married, and being a student compared with being employed (adjusted OR 0·26, 95% CI 0·10–0·68). Perceived risk and symptoms of STIs were motivators for testing whereas misinformation, anticipated stigma, and concern about confidentiality were barriers.

**Interpretation:**

The prevalence of chlamydia or gonorrhoea, or both, was high among youth but only a minority were symptomatic. Therefore most infections would remain untreated without access to STI testing. Provision of education, counselling, and confidentiality are essential to improve uptake and acceptability of STI testing.

**Funding:**

Wellcome Trust.

## Introduction

Globally, 376 million curable sexually transmitted infections (STIs) were recorded in 2016 among people aged 15–49 years.^1^ Although the concerted focus on HIV infection has led to a substantial decline in HIV incidence in many countries, STI programmes have not been effective and the prevalence of STIs globally is increasing.^2^ Adolescents and young people, especially young women, are at particularly high risk of STIs, with higher prevalence than that in older age groups observed in east and southern Africa.^3^

Over the past three decades, syndromic management of STIs has been implemented in resource-limited settings due to lack of laboratory capacity. However, syndromic management has consistently been shown to have very poor sensitivity and specificity, leading to considerable levels of both underdiagnosis and overtreatment.^4^ Crucially, many curable STIs are asymptomatic, but syndromic management requires the presence of symptoms. Therefore, in settings where diagnostic testing is not available, participants with asymptomatic STIs will not receive treatment. The WHO 2016–21 strategy on STIs notes that “investment in point-of-care test development will generate future savings by lowering STI diagnostic and screening costs, and improving case management and detection of asymptomatic STI, thus contributing to lower STI burdens”.^5^ Over the past decade, simpler diagnostic platforms for STIs that do not require sophisticated laboratory infrastructure have been developed. However, data on uptake and acceptability of testing if offered to asymptomatic individuals, particularly among youth, are sparse.

**Research in context**

**Evidence before this study**

We searched MEDLINE for articles published before July 30, 2020, with no language restrictions, with the search terms (variations on and synonyms of) “sexually transmitted infections” AND “testing” AND “uptake” AND “youth” AND “sub-Saharan Africa”. Of the 619 articles identified by this search, there were no studies that assessed uptake of sexually transmitted infection (STI) testing among youth when offered universally in a community-based setting in sub-Saharan Africa. A study published in 2020, investigated self-reported “lifetime STI testing among urban refugee and displaced youth living in informal settlements in Kampala, Uganda. Among young women, predictors of lifetime STI testing were older age, lower sexual activity and pregnancy stigma, and lower food insecurity. Except for older age, predictors were different for young men including higher self-efficacy in condom use, and increased sexual activity and pregnancy stigma. However, lifetime STI testing was self-reported and thus subject to bias and misclassification.

**Added value of this study**

This study is an important addition to a very sparse literature. To our knowledge, it is the first study to implement testing for *Chlamydia trachomatis* (chlamydia) and *Neisseria gonorrhoeae* (gonorrhoea) infections in adolescents in a community-based setting in sub-Saharan Africa and to investigate the cascade from testing to treatment including partner notification. Crucially, uptake of testing and associated factors was assessed after testing was actually offered to youth. Additionally, we found a high prevalence of chlamydia and gonorrhoea yet only a small minority reported the presence of symptoms. The qualitative data from in-depth interviews further contextualised the barriers that youth face in accessing services for STIs including stigma, misinformation, and concerns surrounding confidentiality. We have also showed the difficulties associated with partner notification, and further research is needed to optimise this process.

**Implications of all the available evidence**

This study contributes towards the mounting evidence regarding the limitations of syndromic management and the need to further develop and implement point-of-care diagnostics in resource-limited settings. We have demonstrated the feasibility of a community-based model for STI testing for youth in resource-limited settings. Further innovative strategies for the delivery of STI testing and care will need to be developed to ensure high levels of uptake as point-of-care tests for STIs become more widely available.

Young people experience multiple barriers to accessing sexual and reproductive health (SRH) services. In addition to a lack of availability or knowledge of services, costs and long waiting times as a barrier to access, perceived judgmental attitudes of health-care providers, and concerns surrounding confidentiality result in low uptake of services.^6^, ^7^ Therefore, STI testing strategies will need to account for and address these factors to facilitate optimum uptake and acceptability.

The aims of this study were to investigate the prevalence of *Chlamydia trachomatis* (chlamydia) and *Neisseria gonorrhoeae* (gonorrhoea), and uptake and yield of testing offered as part of an integrated package of SRH and HIV services in community-based settings to youth aged 16–24 years in Harare, Zimbabwe, and to explore facilitators and barriers to uptake of chlamydia and gonorrhoea testing.

## Methods

### Study design and participants

This study was nested within the CHIEDZA trial (Community based interventions to improve HIV outcomes in youth: a cluster randomised trial in Zimbabwe; NCT03719521), a cluster randomised trial investigating the effect of an integrated package of HIV and SRH services for youth in community-based settings on population level HIV prevalence and other health outcomes. The two-arm trial is being done in 24 clusters in three provinces (Harare, Mashonaland East, and Bulawayo), with each province randomised to four intervention and four standard of care (routine, existing services) clusters (1:1). A cluster is a geographically demarcated area containing a primary health-care clinic and a community centre from which services are delivered.

Individuals aged 16–24 years living within an intervention cluster are eligible to receive a package of services including HIV testing, HIV treatment and adherence support, contraception, pregnancy testing, syndromic management of STIs, menstrual health information and products, condoms, and general health counselling. Treatment of STIs and HIV is delivered according to national guidelines. In each cluster, services are delivered once weekly (on the same day each week) by a team of nurses, community health workers, youth workers, and a counsellor, and all services are offered free of cost. The services are configured to be youth-friendly and to ensure confidentiality. The services are provided from 1100 h to 1900 h and operate on an open access basis with no previous appointments required. There is no limit to the number of times an individual can access the service over time. The present study was done in the four intervention clusters in Harare.

Ethical approval for the CHIEDZA trial including the STI study was obtained from the Medical Research Council of Zimbabwe and The London School of Hygiene & Tropical Medicine Ethics Committee. Regarding services offered through the CHIEDZA intervention, verbal consent was sought for chlamydia and gonorrhoea testing. Written informed consent in Shona or English was obtained from participants who were enrolled for the questionnaire and for qualitative interviews. The protocol for the CHIEDZA trial has been previously published.

### Procedures

All individuals accessing CHIEDZA services were non-selectively offered confidential testing for chlamydia and gonorrhoea, regardless of whether they had symptoms. Those who accepted testing provided a first-catch urine sample which was tested within 48 h of collection using the GeneXpert platform (Cepheid, Sunnyvale, CA, USA). Participants were eligible for repeat testing after 6 months. To maintain confidentiality, participants provided a pseudonym and samples were processed using an identification number.

All participants were given the option to pick up their result the following week, and those with a positive result were actively followed up by telephone. If uncontactable, repeat phone calls were made weekly for up to 2 months, after which a particpant was considered lost to follow-up. Individuals who reported STI symptoms at presentation were treated according to national guidelines for syndromic management. If treatment was given on the day of presentation, they were not actively contacted about a subsequent positive chlamydia and gonorrhoea test result. Given that the intervention team delivered services to a cluster once weekly, the shortest possible duration between testing and treatment for asymptomatic participants was 7 days. Partner notification slips were given to those who were treated for an STI, and all partners were offered presumptive treatment regardless of age and whether they resided in the intervention cluster.

Chlamydia and gonorrhoea testing uptake data were regularly reviewed in real-time and discussed at the intervention team's monthly debrief meetings. During the course of the study, the intervention team underwent further training on STIs and written Information, Education and Communication material for participants was developed to enhance uptake of chlamydia and gonorrhoea testing.

Participants accessing CHIEDZA services at the four intervention sites in Harare between June 10 and July 16, 2019, were consecutively enrolled to complete an interviewer-administered questionnaire to understand factors associated with uptake of chlamydia and gonorrhoea testing. The questionnaire recorded sociodemographic and sexual behaviour data, and included a mixture of open-ended and closed-ended questions to assess knowledge of STIs and to solicit opinions about STI service delivery including partner notification.

A separate purposively selected sample was also recruited to participate in in-depth interviews to explore understanding of STIs and perceived facilitators and barriers to chlamydia and gonorrhoea testing. Participants were selected to provide roughly equal representation of men and women and those who accepted and declined chlamydia and gonorrhoea testing. Interviews were done by a trained interviewer (not involved in service delivery) at a secluded spot outside the community centre, in either Shona or English depending on participant preference. Interviews were done after a participant had completed their consultation with a health worker and were continued until data saturation was achieved. All interviews were audio-recorded and translated and transcribed by the interviewer.

The primary outcome of this study was uptake of STI testing. Secondary outcomes included prevalence of chlamydia and gonorrhoea, and yield of testing.

### Statistical analysis

Sample size calculations to investigate chlamydia and gonorrhoea testing uptake assumed a ratio of 1:2 between those accepting and declining STI testing. Given the dearth of data on uptake of chlamydia and gonorrhoea testing in similar settings, this ratio was informed by the range of uptake reported in high-income countries^8^ together with the fact that testing was offered within a package of youth-friendly services. Assuming a prevalence of a risk factor of 20% among those declining testing and a type I error rate of 5%, a sample size of 204 (68 accepting testing) would provide 80% power to detect an odds ratio (OR) of 2·5.

The prevalence of chlamydia and gonorrhoea and uptake and yield of testing were recorded, and the proportion of participants with positive tests who received treatment, and time to treatment were calculated.

Factors associated with uptake of STI testing were investigated using multivariate logistic regression. We used a hierarchical conceptual approach with three levels: (1) sociodemographic, (2) behavioural, and (3) other more proximal factors (appendix p 1).^9^, ^10^ Age and sex were considered a priori confounders for each model. For level 1, all age-adjusted and sex-adjusted sociodemographic factors that met a p value cutoff of less than 0·10 were included in the multivariate model for level 1. Those factors that remained associated at less than 0·10 became the core level 1 factors. For level 2, all core level 1-adjusted behavioural factors that met a p value cutoff of less than 0·10 were included in the multivariate model for level 2, together with the core level 1 factors. Associations with level 3 factors were determined in a similar way. If factors that were restricted by reported penile–vaginal sex or sex met the p value cutoff and we wanted to use the factor to adjust other variables, a reference category was added to the variable so that there would be an unrestricted adjusted model. For example, for factors restricted to participants who reported having had penile–vaginal sex, a reference category of no penile–vaginal sex was added to the variable when included in the adjusted model.

Qualitative data were analysed using thematic analysis on the basis of the following themes: facilitators and barriers to chlamydia and gonorrhoea testing uptake and facilitators and barriers to partner notification. Following familiarisation with data, codes were generated on the basis of these themes and subthemes emerging from the transcripts. NVivo 12 (QSR International) was used to assist with coding transcripts. Themes and coding were iteratively reviewed and refined. Data analysis was done with STATA version 15.0.

### Role of the funding source

The funder of the study had no role in study design, data collection, data analysis, data interpretation, or writing of the report. All authors had full access to all the data in the study and accept responsibility for the decision to submit for publication.

## Results

**Figure 1:**
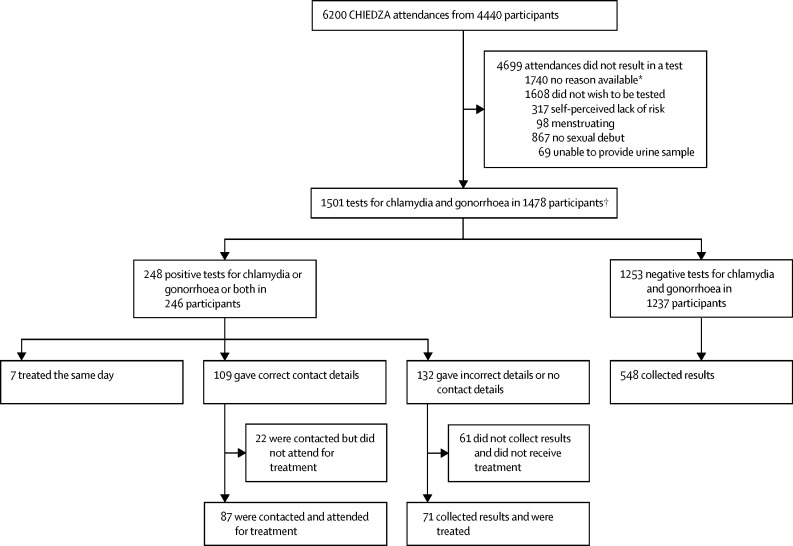
Study profile *Reasons for lack of uptake documented from Sept 3, 2019, onwards. †23 participants tested twice during the study period.

Between June 1, 2019, and Jan 31, 2020, there were 6200 attendances to CHIEDZA services by 4440 young people who met the eligibility criteria for chlamydia and gonorrhoea testing. 3473 (78·2%) were women and 967 (21·8%) were men, and the median age was 20·3 years (IQR 17·9–22·8). Of the 6200 attendances, 4699 (76%) were not tested for reasons including refusal, self-perceived lack of risk, menstruation, not yet sexually active, or unable to provide a urine sample (figure 1).

**Figure 2:**
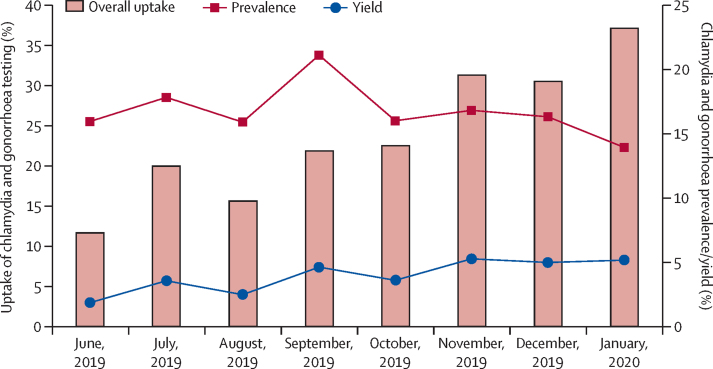
Prevalence of chlamydia and gonorrhoea, and uptake and yield of testing

In total, 1501 STI tests (24·2%) were done of the 6200 attendances, in 1478 people, with 23 people tested twice over the study period at an interval of at least 6 months, equating to an uptake of at least one chlamydia and gonorrhoea test of 33·3% (95% CI 31·9%–34·7%; figure 1). The uptake in men was 30·4% (294 of 967) and in women was 34·1% (1184 of 3473). The uptake of testing among eligible attendees increased over time from 11·7% (94 of 806) in the first month to 37·1% (287 of 773) in month eight (figure 2). The chlamydia and gonorrhoea prevalence among those tested was 16·5% (95% CI 14·7–18·5; 248 of 1501). The prevalence of these STIs was lower in men than in women (30 [10%] of 300 *vs* 218 [18·2%] of 1201, p=0·0010; table 1). Of the 23 participants who underwent testing at two timepoints, two tested positive on both occasions and 16 tested negative both times. The overall yield of testing was 4.0% (95% CI 3·5–4·5; 248 of 6200).

**Table 1:** Prevalence of chlamydia and gonorrhoea by age group and sex

	**Total (n=1501)**		**Men**						**Women**					
	Number of participants	95% CI	16–20 years (n=187)		21–24 years (n=113)		Total for age 16–24 (n=300)		16–20 years (n=420)		21–24 years (n=781)		Total for age 16–24 (n=1201)	
			Number of participants	95% CI	Number of participants	95% CI	Number of participants	95% CI	Number of participants	95% CI	Number of participants	95% CI	Number of participants	95% CI
Chlamydia	200 (13·32%)	11·64–15·15	8 (4·28%)	1·86–8·26	14 (12·39%)	6·94–19·91	22 (7·33%)	4·65–10·89	68 (16·19%)	12·80–20·06	110 (14·08%)	11·72–16·72	178 (14·82%)	12·80–16·96
Gonorrhoea	34 (2·27%)	1·57–3·15	4 (2·14%)	0·59–5·39	3 (2·65%)	0·55–7·56	7 (2·33%)	0·94–4·75	13 (3·10%)	1·66–5·23	14 (1·79%)	0·98–2·99	27 (2·25%)	1·49–3·25
Chlamydia and gonorrhoea	14 (0·93%)	0·51–1·56	0	..	1 (0·88%)	0·02–4·83	1 (0·33%)	0·00–1·84	4 (0·95%)	0·26–2·42	9 (1·15%)	0·53–2·18	13 (1·08%)	0·58–1·84

Data are n (%) unless otherwise specified. All 95% CIs presented are for percentages.

Of 248 participants with a positive STI test, 165 (67%) were treated. Seven (3%) participants were symptomatic on presentation and following examinations were treated on the same day using syndromic treatment guidelines. Five (2%) were female participants with vaginal discharge syndrome (three with gonorrhoea only, one with chlamydia only, and one with both infections), and two (<1%) were male participants with urethral discharge syndrome (one with gonorrhoea only and one with chlamydia only). Correct traceable contact details had only been provided by 109 (44%) of the 248 participants, and of these 87 (80%) attended for treatment. Of the 132 participants who were uncontactable, 71 (54%) presented to collect results and were treated. The median time between testing and treatment in the 158 participants who were not treated at presentation was 7 days (IQR 7–21). Of the 1253 participants who had negative test results (and were therefore not contacted), 548 (43·7%) attended to collect their results within two months of testing. Overall, 22 partners were treated through the partner notification process.

A total of 257 participants were recruited between June 10, and July 16, 2019, to investigate factors associated with uptake of chlamydia and gonorrhoea testing. Participants were broadly representative of those in the CHIEDZA study; the median age was 20 years (IQR 18–22) and 161 (63%) were women (table 2). 84 (33%) participants used condoms regularly and 80 (31%) used condoms at last sexual intercourse. Being a woman, older (age 21–24 years compared with age 16–20 years), married (compared with being single or having a girlfriend or boyfriend), employed (compared with being a student), having at least one sexual partner in the previous 12 months, having current STI symptoms, not using a condom at last sexual intercourse and lower levels of reported condom use, higher levels of perceived risk of an STI, and having had at least one pregnancy were associated with uptake of testing in the unadjusted analysis. After adjusting for age, sex, occupation and wwmarital status, current STI symptoms remained strongly associated with accepting chlamydia and gonorrhoea testing (adjusted OR 14·79, 95% CI 1·66–132·07). Having a boyfriend or girlfriend (adjusted OR 0·19, 95% CI 0·087–0·43) or being single (adjusted OR 0·33, 95% CI 0·13–0·84) compared with being married, and being a student compared with being employed (adjusted OR 0·26, 95% CI 0·10–0·68) were negatively associated with chlamydia and gonorrhoea testing uptake.

**Table 2:** Multivariate analysis of factors affecting uptake of STI testing

		**STI testing uptake**	**Unadjusted OR (95% CI)**	**p value**	Adjusted OR*(95% CI)	**p value**
**Sociodemographic factors (level 1)**						
Age, years						
	16–20	37/148 (25%)	1 (ref)	0·0003	1 (ref)	0·34
	21–24	51/109 (47%)	2·64 (1·55–4·48)		1·37 (0·72–2·59)	
Sex						
	Male	24/96 (25%)	1 (ref)	0·017	1 (ref)	0·79
	Female	64/161 (40%)	1·98 (1·13–3·46)		0·92 (0·47–1·78)	
Occupation						
	Employed	18/35 (51%)	1 (ref)	0·0001	1 (ref)	0·019
	Unemployed	54/127 (43%)	0·70 (0·33–1·48)		0·53 (0·22–1·26)	
	Student	16/95 (17%)	0·19 (0·08–0·45)		0·26 (0·10–0·68)	
Education						
	Post-secondary	5/20 (25%)	1 (ref)	0·50	1 (ref)	0·98
	Secondary	76/221 (34%)	1·57 (0·55–4·49)		0·90 (0·26–3·12)	
	Primary	7/16 (44%)	2·33 (0·57–9·60)		0·84 (0·16–4·45)	
Marital status						
	Married	41/63 (65%)	1 (ref)	<0·0001	1 (ref)	0·0003
	Partner	30/135 (22%)	0·15 (0·08–0·30)		0·19 (0·087–0·43)	
	Single	17/59 (29%)	0·22 (0·10–0·47)		0·33 (0·13–0·84)	
**Sexual behaviour factors (level 2)**						
Ever had oral sex†						
	No	54/176 (31%)	1 (ref)	0·077	1 (ref)	0·52
	Yes	34/81 (42%)	1·63 (0·95–2·82)		1·24 (0·64–2·41)	
Ever had anal sex†						
	No	86/248 (35%)	1 (ref)	0·45	1 (ref)	0·47
	Yes	2/9 (22%)	0·54 (0·11–2·65)		0·54 (0·10–2·92)	
Ever had penile–vaginal sex						
	No	11/77 (14%)	1 (ref)	<0·0001	1 (ref)	0·099
	Yes	77/180 (43%)	4·49 (2·22–9·06)		2·17 (0·86–5·45)	
Age at sexual debut‡, years						
	>16	57/129 (44%)	1 (ref)	0·54	1 (ref)	0·32
	≤16	20/51 (39%)	0·81 (0·42–1·58)		1·49 (0·67–3·31)	
Number of sexual partners in past 12 months§						
	0	12/83 (14%)	1 (ref)	<0·0001	1 (ref)	0·13
	1	54/106 (51%)	6·14 (2·99–12·63)		2·50 (0·91–6·87)	
	≥2	22/68 (32%)	2·83 (1·28–6·27)		2·79 (0·97–8·03)	
New sexual partner in past 3 months						
	No	75/208 (36%)	1 (ref)	0·21	1 (ref)	0·19
	Yes	13/49 (27%)	0·64 (0·32–1·28)		0·57 (0·24–1·31)	
Condom use at last sex‡						
	Yes	25/80 (31%)	1 (ref)	0·0056	1 (ref)	0·37
	No	52/100 (52%)	2·38 (1·29–4·41)		0·66 (0·26–1·66)	
Condom use‡						
	Always or mostly	27/84 (32%)	1 (ref)	0·0074	1 (ref)	0·37
	Sometimes or rarely or never	50/96 (52%)	2·29 (1·25–4·21)		0·66 (0·27–1·64)	
Money or gifts for sex						
	No	85/249 (34%)	1 (ref)	0·84	1 (ref)	0·89
	Yes	3/8 (38%)	1·16 (0·27–4·96)		1·11 (0·23–5·37)	
Current contraception use (barrier methods excluded)‡¶						
	None	19/43 (44%)	1 (ref)	0·31	1 (ref)	0·94
	Oral contraceptive pill	27/42 (64%)	2·27 (0·95–5·44)		0·95 (0·31–2·90)	
	Depot injection	3/6 (50%)	1·26 (0·23–6·98)		0·55 (0·084–3·61)	
	Implant	10/17 (59%)	1·80 (0·58–5·63)		0·94 (0·26–3·37)	
	Intrauterine contraceptive device	0/1	..		..	
Circumcised‖						
	Yes	8/32 (25%)	1 (ref)	0·48	1 (ref)	0·61
	No	8/44 (18%)	0·67 (0·22–2·02)		0·74 (0·22–2·43)	
**Proximal factors (level 3)**						
Number of pregnancies¶						
	0	17/85 (20%)	1 (ref)	<0·0001	1 (ref)	0·64
	1	25/42 (60%)	5·88 (2·61–13·27)		1·41 (0·41–4·79)	
	≥2	22/34 (65%)	7·33 (3·04–17·71)		0·89 (0·19–4·10)	
Current STI symptoms‡						
	No	66/168 (39%)	1 (ref)	0·0073	1 (ref)	0·016
	Yes	11/12 (92%)	17·00 (2·14–134·78)		14·79 (1·66–132·07)	
Perceived STI risk						
	Unlikely or no risk	65/214 (30%)	1 (ref)	0·0015	1 (ref)	0·45
	Very likely or likely	22/36 (61%)	3·60 (1·73–7·48)		1·33 (0·55–3·23)	
	Unsure	1/7 (14%)	0·38 (0·045–3·24)		0·30 (0·031–2·87)	
Self-reported HIV status						
	Negative	85/253 (34%)	1 (ref)	0·13	1 (ref)	0·67
	Positive	3/4 (75%)	5·93 (0·61–57·86)		1·68 (0·16–17·60)	

Data are n/N (%), unless otherwise specified. STI=sexually transmitted infection. OR=odds ratio.

* Sociodemographic factors adjusted for age, sex, occupation, and marital status (sociodemographic core set); sexual behavioural factors adjusted for sociodemographic core set and if ever had penile–vaginal sex; proximal factors were adjusted for sociodemographic core set, if ever had penile–vaginal sex, and presence of current symptoms.

† Combined giving or receiving.

‡ Question restricted to participants who reported having ever had penile–vaginal sex (n=180).

§ Because of evidence of collinearity “number of sexual partners in past 12 months” was not adjusted for “ever had penile–vaginal sex”.

¶ Question restricted to female participants (n=161).

‖ Question restricted to male participants (n=76).

Knowledge about STIs was highly variable. Of the 257 participants who completed the questionnaire, 231 (90%) knew that STIs could be prevented by condom use, and 217 (84%) knew that these infections could facilitate HIV transmission. However, 141 participants (55%) could not name any complications of STIs (table 3). Furthermore, 26 (10%) participants thought that insect bites and 25 (10%) thought that sharing clothes or towels could transmit STIs.

**Table 3:** Knowledge of STIs and preferences for STI testing

		**Number of participants (n=257)**
**Knowledge of STIs**		
STI symptoms*		
	Genital ulcers	164 (64%)
	Genital discharge	99 (39%)
	Rash	99 (39%)
	Groin swelling	46 (18%)
	Dysuria	45 (18%)
	Itch	42 (16%)
	Abdominal pain	24 (9%)
	Warts	15 (6%)
	Weight loss	9 (4%)
	Hair loss	9 (4%)
	Haematuria	8 (3%)
	Fever	7 (3%)
	Red lips	5 (2%)
	Erectile dysfunction	1 (<1%)
	Unsure	18 (7%)
Complications of STIs*		
	Infertility	72 (28%)
	Cancer	22 (9%)
	HIV or AIDS	19 (7%)
	Blindness	7 (3%)
	Testicular pain or swelling	11 (4%)
	Miscarriage or ectopic pregnancy	5 (2%)
	Death	3 (1%)
	Pelvic inflammatory disease	2 (1%)
	Loss of genitalia	3 (1%)
	Neonatal disabilities	1 (<1%)
	Unsure	141 (55%)
Modes of transmission of STIs†		
	Sex	256 (100%)
	Infected blood	189 (74%)
	Vertical	151 (59%)
	Kissing	47 (18%)
	Insect bite	26 (10%)
	Sharing clothes or towels	25 (10%)
	Shaking hands	0
**Preferences for service delivery**		
Treatment-seeking behaviour for STI symptoms*‡		
	Attend clinic	170/180 (94%)
	Attend pharmacy	10/180 (6%)
	Wait for symptoms to resolve	2/180 (1%)
	Get advice from family or friends	13/180 (7%)
	Attend a traditional healer	1/180 (1%)
	Consult a private doctor	2/180 (1%)
Preferred STI testing location*		
	Community-based non-clinic setting	190 (74%)
	Clinic	168 (65%)
	School or college	30 (12%)
	Private doctor	13 (5%)
	Other§	5 (2%)
Preferred methods for receiving STI test results†		
	In person	233 (91%)
	Phone call	58 (23%)
	Short messaging service (SMS)	43 (17%)
	Post	1 (<1%)
**Partner notification**		
Reasons for not notifying partner if diagnosed with STI*‡		
	Partner might leave	92/180 (51%)
	Accusation of infidelity	27/180 (15%)
	Violence from partner	22/180 (12%)
	Others might find out	18/180 (10%)
	Embarrassment	16/180 (9%)
	Not the index's responsibility	5/180 (3%)
	Other¶	8/180 (4%)
Preferred methods for partner notification†‡		
	Referral by index	126/180 (70%)
	Referral by provider	65/180 (36%)
	Direct delivery of medication to partner by index	1/180 (1%)
	Would not contact	15/180 (8%)
If diagnosed with STI, would inform partner‡‖		
	Yes	142/180 (79%)
	No	36/180 (20%)
	Unsure	2/180 (1%)
	Never had sex	77/180 (43%)
Able to contact all sexual partners of past 3 months‡‖		
	Yes	166/180 (92%)
	No	11/180 (6%)
	Unsure	3/180 (2%)
	Never had sex	77/180 (43%)

Data are n (%), or n/N (%) and include all answers given by participants. STI=sexually transmitted infection.

* Open-ended question with no options provided.

† Closed-ended question, but responses could be elicited in more than one category.

‡ Question restricted to participants who said they had penile–vaginal sex (n=180).

§ Home-based testing, n=2; church, n=1; pharmacy, n=1; workplace, n=1.

¶ Not able to contact partner, n=3; would not see partner again, n=2; might be accused of testing positive for HIV, n=1; partner might worry, n=2.

‖ Closed-ended question for which response was confined to a single category.

Regarding testing procedures, all 96 male participants and 154 (96%) of 161 female participants stated that they would prefer urine samples to a genital swab. The most preferred option for provision of STI testing services was in a community-based, non-clinical setting and only a minority identified schools as a venue where STI services should be offered (table 3). Additionally, 233 (91%) participants preferred to receive STI test results in person; 58 (23%) selected telephone and 43 (17%) selected text as options for receiving test results.

Although 166 (92%) of 180 participants said they would be able to contact sexual partners from the past 3 months, only 142 (79%) said that they would inform them about a positive STI test result. Notably, 92% (100 of 109) of female participants versus only 59% (42 of 71) of male participants said they would inform a partner about a positive STI result. The need to treat the partner for the partner's sake (151 [84%]) and to prevent reinfection (65 [36%]) were the most common reasons noted for notifying partners. Reasons for not telling a partner about a positive STI result were fear of the partner leaving (92 [51%]), being blamed for the STI (27 [15%]), and risk of violence (22 [12%]). Regarding the referral process itself, 126 (70%) participants preferred notifying partners themselves (patient referral). However, 65 (36%) selected provider referral, whereby a health-care worker informs the contact directly, as an appropriate option for partner notification.

26 young people participated in in-depth interviews. Participants were aged 16–24 years (median 19), 16 (62%) were women, and ten (38%) were men. Of these, 12 had agreed to testing, 12 had declined testing, and two were unaware of testing being available. Emergent themes and supporting quotes are presented in the appendix (p 2).

STI testing uptake was motivated by presence of symptoms and potential treatment if they tested positive. Additionally, participants' decision to be tested was driven by their perceived risk, based on their own and their partner's sexual behaviour.

Stigma was a key barrier factor to uptake of STI testing. Many feared family and community members finding out and were worried “about what other people might say about you” and being ostracised by their familes if found to be sexually active. Anticipated stigma alongside perceived lack of confidentiality among health-care workers was not only a barrier to uptake of testing, but influenced young people's choice of health-care providers. Participants stated they would choose either “the furthest” hospital from them or a private clinic, so they wouldn't “be recognised”. There were also misconceptions that impeded uptake of STI testing, including the belief that circumcision conferred complete protection from STIs, that menstruation precluded STI testing, and that HIV testing covered other STIs. Notably, there was much less concern about curable STIs than about HIV and absence of symptoms deterred participants from testing.

Participants were aware that if they had an STI, their partner might also have an STI and should be treated to protect them and to prevent reinfection. Although participants acknowledged a general need for honesty, telling partners was perceived to be a particular challenge.

## Discussion

We found a high prevalence of chlamydia and a moderate prevalence of gonorrhoea among youth offered testing in community-based settings, only a minority of whom had symptoms. One in six youth who took up testing tested positive for chlamydia or gonorrhoea, or both. Importantly, testing was not targeted at those with high risk behaviours or symptoms. The uptake of testing improved over time, and uptake and prevalence of the STIs was higher in female participants than in male participants. The prevalence of chlamydia and gonorrhoea was high in comparison to that reported in two previous systematic reviews of STI prevalence among women in sub-Saharan Africa.^3^, ^11^ These findings emphasise the substantial STI epidemic in southern Africa and the unmet need for STI services, particularly among youth who consistently have a higher prevalence of STIs than do older age groups.^4^, ^12^, ^13^ Chlamydia was more prevalent than gonorrhoea in this group of mostly asymptomatic youth. However, in previous studies in Zimbabwe, gonorrhoea was more common than chlamydia among men and women presenting with urethral and vaginal discharge.^14^, ^15^

The availability of simple diagnostic platforms provides an opportunity to move away from syndromic management.^16^ The GeneXpert platform is a closed cartridge system that does not require sophisticated laboratory infrastructure or expert skills and is widely available in sub-Saharan Africa. Given that a single dose treatment is available for STIs such as chlamydia and gonorrhoea, point-of-care STI diagnostics paired with immediate treatment would have an impact on STI control.^17^ However, in addition to the high cost, the need for a continuous power supply and the time to result (90 min) preclude the use of the Xpert CT/NG platform as a true point-of-care test.^18^ In our study, the earliest available results were 1 week post-testing, which might have resulted in lower treatment rates of those diagnosed with an STI.

Diagnostic testing will only be effective in curbing the STI epidemic if accessible, acceptable delivery strategies are developed. Youth face particular sociocultural and structural barriers to accessing sexual and reproductive health services including poor health literacy, stigma, shame, judgmental attitudes of health-care providers, and the need for guardian consent. Globally health facility usage rates among youth remain low.^6^ Most participants stated they would access a clinic if they developed STI symptoms, but this may reflect their familiarity with a clinic setting. Only a minority favoured educational institutions for STI service delivery due to concerns about confidentiality, a finding also raised previously in a study of Kenyan female adolescents.^19^ STI testing in a community-based setting was the most commonly chosen option by participants. This might be because chlamydia or gonorrhoea testing was offered as part of an integrated package of youth-friendly HIV and sexual and reproductive services codesigned with youth to optimise accessibility and acceptability. However, given that participants were recruited while accessing a community-based sexual and reproductive service, these results might not be representative of youth in the wider community and desirability bias may have influenced responses.

Uptake of chlamydia and gonorrhoea testing in this study was initially low but increased three-fold from 12% to 37% over the 8 month study period. This increase is probably due to health-care providers gaining more experience and confidence in providing STI services, as well as the availability of Information, Education and Communication material for participants. Modelling studies indicate that uptake is the key factor in determining the effect of screening programmes on prevalence of chlamydia.^20^ Therefore, increased uptake must be achieved and evidence from high-income countries has shown that large amounts of effort and changes to practice are often required to achieve high coverage.^8^ Although young people are regularly exposed to information about HIV through school programmes and media campaigns, much less attention has been paid to curable STIs.^21^, ^22^, ^23^ We found low levels of knowledge about STI risks, symptoms, and complications, a finding also reported by other studies from sub-Saharan Africa.^24^ This has implications for uptake of STI testing as individuals who are asymptomatic might not realise they are infected and could decline testing. Additionally, only 44% of participants noted condom use at last sexual intercourse, showing the need to continue emphasising condom use. Having symptoms was significantly associated with uptake of STI testing on multivariate analysis. However, only a minority of participants had symptoms, and being symptomatic was a poor predictor for having chlamydia or gonorrhoea. Previous studies have shown similar findings, and support the use of an unselected approach to STI testing that is not dependent on symptoms.^4^

In addition to a lack of information, prevailing misinformation was a barrier to STI testing. A number of participants were unaware of the difference between HIV and other STIs, and felt protected from other STIs if they had been tested for HIV. An additional barrier to uptake of testing was fear of a positive test result and consequent stigma. Similar results were reported in a study of adolescent girls from Kenya.^19^ These findings underscore the importance of providing adequate and age-appropriate information to inform decision making and counselling to accompany testing.

Combining STI testing with effective follow-up strategies is crucial to ensure that those diagnosed are treated. Despite active and confidential follow-up, a third of participants with a positive test were lost to follow-up. Only 44% of participants provided correct traceable contact details, which might be related to concerns regarding confidentiality. However, about half of those with a positive test result who were uncontactable, and a similar proportion of those with a negative test result, returned to collect their result spontaneously, demonstrating their interest in their test results.

Partner notification is an important component of STI control as treatment of partners can prevent reinfection of the index patient, break the cycle of transmission, and reduce infection burden.^5^ The yield of partner notification in our study was very low with less than 10% of partners attending for treatment. We cannot be certain that some partners did not attend alternative facilities for follow-up and treatment, but the low rates attending study sites indicate the challenges associated with partner notification.

A systematic review investigating the acceptability and efficacy of partner notifications in sub-Saharan Africa showed that 25% (range 0–77) of partners notified by direct patient referral attended services for evaluation or were treated. Provider referral (69%) and expedited partner treatment (84%) was more successful than direct patient referral.^25^ Patient referral was the most popular method of partner notification in our study, but about a third of participants also selected provider referral as an option for partner notification. Providing multiple options could improve the success of partner notification, but is not the only consideration. Concerns about being accused of infidelity and fear of violence or the relationship ending impede individuals from informing their partners,^26^, ^27^ and emphasise the importance of counselling and support for both indexes and their partners.

The strengths of the study were the assessment of a large number of participants along the whole cascade of care from testing to treatment and partner notification, the moderate sample size for the regression analyses, the offer of unselected testing and inclusion of men, and the use of mixed research methods to investigate the facilitators and barriers to chlamydia and gonorrhoea testing. The study was done in four urban communities in Harare, Zimbabwe, which limits the generalisability of our findings. Given the stigma surrounding sex, under-reporting of sexual activity, particularly on reporting of socially proscribed behaviours such as transactional sex, might have occurred. We used urine samples, which are slightly less sensitive than vaginal swabs.^28^ Vaginal swabs were not available as an option for women who could not provide urine samples. Additionally, we did not offer testing for other common genital infections such as *Trichomonas vaginalis* or bacterial vaginosis. Finally, some questions on preferences for service delivery were based on hypothetical scenarios, which might have affected the validity of responses. For example, the majority of women preferred urine samples but they had no experience of vaginal swabs. Studies from sub-Saharan Africa show that vaginal swabs are highly acceptable and a systematic review from 2015 found that women found vaginal swabs marginally more acceptable than urine samples.^29^

Overall, our study found a high prevalence of chlamydia and gonorrhoea among youth who were offered unselective testing in a community-based setting. Potential areas for improvement include the inclusion of rapid tests for *T vaginalis* and bacterial vaginosis, allowing for a more comprehensive service. Increased regular testing might be required for young people at particularly high risk of STIs. Strategies to improve follow-up of participants and partner notification are vital to ensure treatment of STIs and the interruption of chains of transmission. Development of affordable point-of-care tests for STIs will help to improve rates of treatment following diagnosis. As availability of diagnostics increases, innovative strategies for delivery of STI testing and care that address the barriers to access and uptake will need to be developed. The lessons learned from HIV testing and treatment programmes such as provision of information and counselling and stigma reduction can serve as a template for designing STI service models. Importantly, given the wide availability of HIV services, meaningful integration of STI services, and more generally sexual and reproductive health services, can create synergies by capitalising on existing infrastructure, knowledge, and skills.

## Data sharing

Individual, anonymised participant data and a data dictionary will be available through The London School of Hygiene & Tropical Medicine repository (Data Compass) 12 months after publication of results.
