# Supplementary material for: Uptake of and factors associated with testing for sexually transmitted infections in community-based settings among youth in Zimbabwe: a mixed-methods study
Source: Lancet Child Adolesc Health. 2021 Feb;5(2):122–32. doi: 10.1016/S2352-4642(20)30335-7 (PMC7818532; doi:10.1016/S2352-4642(20)30335-7)
Supplement: Supplementary appendix [file mmc1.pdf]

THE LANCET
Child & Adolescent Health

Supplementary appendix

This appendix formed part of the original submission and has been peer reviewed. We post it as supplied by the authors.

Supplement to: K Martin, I D Olaru, N Buwu, et al. Uptake of and factors associated with testing for sexually transmitted infections in community-based settings among youth in Zimbabwe: a mixed-methods study. *Lancet Child Adolesc Health* 2021; published online Jan 6. http://dx.doi.org/10.1016/S2352-4642(20)30335-7.

Supplementary figure 1: Conceptual hierarchical framework of factors associated with uptake of CT/NG testing

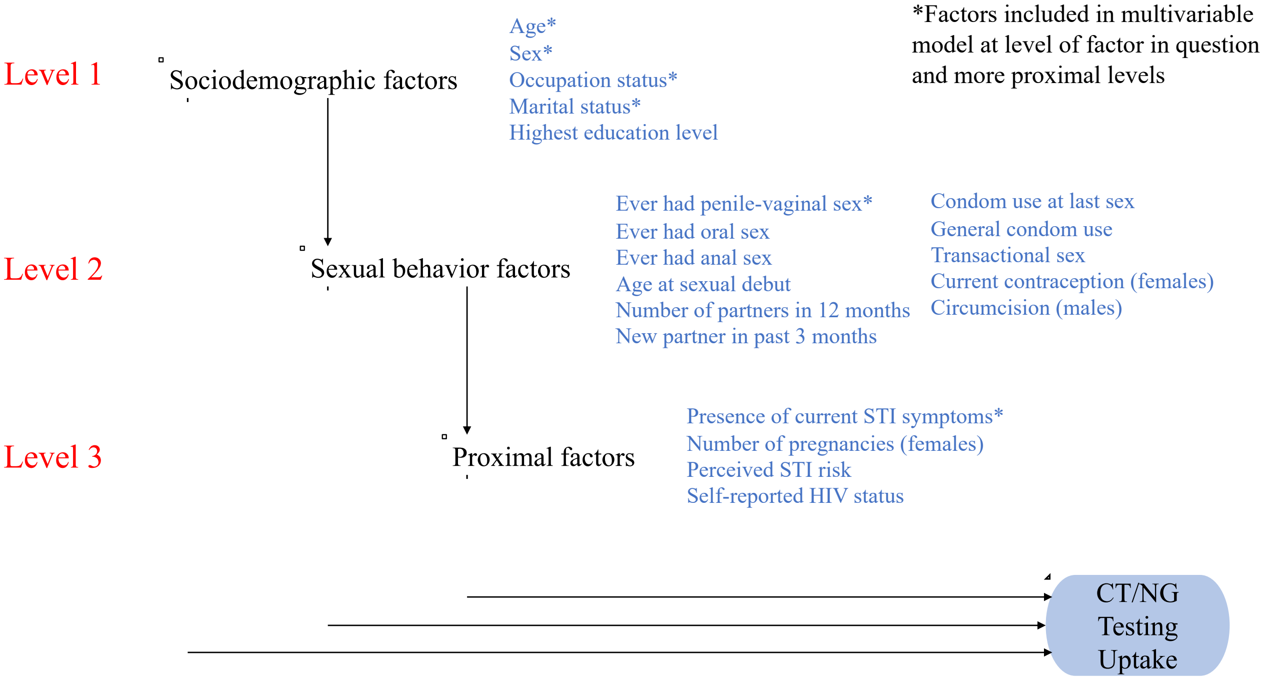

Supplementary table 1: Themes and supporting quotes from interviews

Themes	Quote
Facilitators of STI testing	
**Presence of symptoms**	*"And as for STI testing, I think if you have something suspicious… you should just go and get tested"* (17-year-old female, accepted testing)<br><br>*"I had sores a few months back and they just finished on their own so I just wanted to check if I had it"* (24-year-old female, accepted testing)
**Perceived high risk due to own or partner's behaviour**	*"My husband stays in South Africa, so I won't know what he gets up to"* (23-year-old female, accepted testing)<br><br>*"I don't trust the girl I am having unprotected sex with. She seems promiscuous and I didn't know much about her because she recently started attending our school."* (17-year-old male, accepted testing)<br><br>*"I heard an STI can take long to show symptoms so I thought I should get tested since I have had unprotected sex before."* (23-year-old female, accepted testing)
**Knowing one's status**	*"Since I am a girl it's good that I know if I have an STI for when I get pregnant, so I don't harm the baby during pregnancy."* (20-year-old female, declined testing)<br><br>*"It's actually important and useful because you might have it and yet the symptoms have not started to show, so just knowing is good."* (16-year-old female, declined testing)<br><br>*"I would rather be tested first so that I know what I am having and then be treated."* (24-year-old female, accepted testing)<br><br>*"I would come here and get tested and find out if what I'm feeling are the symptoms of an STI or not. Then I know where I stand and if the problem can be solved."* (20-year-old female, declined testing)
**Importance of early treatment**	*"I think it's a good thing because it will help you know if you have an STI or not and you are able to receive treatment on time rather than not get tested and it spreads and becomes untreatable."* (24-year-old female, accepted testing)<br><br>*"For example, if I immediately see that this is what has happened, I can quickly get treatment so that it goes away immediately. We wouldn't want to it to show to people that I am sick."* (21-year-old female, accepted testing)
Barriers to STI testing	
**Perceived lack of confidentiality**	*"At my age, I would look for the furthest one [hospital] because like right now if I think I have symptoms, obviously I can't go there because the people who work there know my parents."* (17-year-old male, accepted testing)<br><br>*"Yeah, [a] private clinic is better because maybe you don't want to be recognised. Most of the time people don't want people to know that is what is going on"* (18-year-old female, declined testing)
**Stigma surrounding STIs and STI testing**	*"On receiving a positive [STI] result You might isolate yourself from society and all that. Think about what other people might say about you."* (21-year-old male, declined testing)<br><br>*"Fear that if I tell them [parents], like for my age they might say I started doing this at a young age and they might even ask me to pack my bags and tell me to leave the house."* (16-year-old female, declined testing)<br><br>*"I didn't accept it like easily because I thought that I am a clean girl, I thought that I am very smart… I showed my Mum I have this discharge and then she was like "it's an STI" so I didn't accept it, I was like no, no I can't get an STI… It's not easy to accept but you have to accept it. As for me I cried, I suffered a lot."* (17-year-old female, accepted testing)

**Anxiety associated with receiving a positive result**	*"Uh from my thinking, I think if you find out you are positive, like you see me I am fit, but that thing called stress is not good at all."* (17-year-old male, accepted testing)<br><br>*"Honestly speaking, I was afraid… That's the truth. I was afraid. But I was dumb for it. I would have done it. But then, since because I was afraid, I couldn't do it."* (23-year-old male, declined testing)
**Lack of symptoms and concern about STIs**	*"It's better that one knows their [HIV] status then you get into the other things [other STIs] that don't worry you so much"* (20-year-old male, declined testing)<br><br>*"I haven't seen any signs that I might have STIs, so I think I'm relaxed about the issue."* (21-year-old male, declined testing)<br><br>*"I don't have any symptoms so didn't think I need to be tested"* (20-year-old female, declined testing)
**Misperceptions about STIs and STI testing**	*"I thought if someone has got an STI, it is obvious that that person is HIV positive… So…when I got tested in STI and I uh, it was positive, I then got tested many times [for HIV] because I was like, oh so I am also HIV positive. But when I got tested, I realized that, a person can be STI positive and HIV negative."* (17-year-old female, accepted testing)<br><br>*"I cannot get an STI because the foreskin was removed. So, there is no way I can have an STI. That's what they explained to me"* (21-year-old male, declined testing)<br><br>*"I tested last week for HIV so didn't feel need to test"* (17-year-old male, declined testing)<br><br>*"I was on my period so wasn't sure if I should [get tested]"* (21-year-old female, declined testing)
Facilitators of Partner Notification	
**Need for partner to be tested or treated**	*"Yes, it's important because you will be protecting your sexual partner from the risks."* (24-year-old female, accepted testing)<br><br>*"Maybe they are the one who would have given me that problem or… I would have already given it to them so I would be helping on the other side so that she too can get tested"* (23-year-old male, declined testing)
**Prevention of re-infection**	*"Yes, I would tell them that's what has been diagnosed. Because if he doesn't get treated for the disease then it will carry on and I will be re-infected"* (22-year-old female, accepted testing)
**General need for honesty**	*"Yeah. It wouldn't be fair to keep it a secret, you know."* (21-year-old male, declined testing)<br><br>*"It's so that he doesn't worry about where he got it but to know he got it from me"* (20-year-old female, accepted testing)
Barriers to Partner Notification	
**Accusations of infidelity**	*"You may be afraid that he will break up with you and he might say that he doesn't have an STI problem and that I could have gotten it from somewhere else."* (20-year-old female, declined testing)<br><br>*"What I think is that like me I have a boyfriend and maybe I also have someone I am having sex with… for me to tell him, he might ask where did I get it from?"* (22-year-old female, accepted testing)
**Fear of relationship ending**	*"Maybe you will be afraid that he might not be the one who has infected you. Maybe I gave it to him so this can end the relationship" (16-year-old female, declined testing)*<br><br>*"They might leave you; ah they might say "you have this disease so we can't be together""* (22-year-old female, accepted testing)
**Risk of others finding out**	*"Maybe the person [is] not one that can keep a secret. They might go around spreading it to everyone. Sometimes it could be that this person is the one that infected you, we wouldn't know."* (16-year-old male, unaware of testing)

	"Most of the time you are afraid that they will leave you or they can go around telling people though it also depends what kind of boyfriend you have." (18-year-old female, accepted testing)
